# Effect of 5-Aminolevulinic Acid Photodynamic Therapy with Transfer Factor Capsules in the Treatment of Multiple Plantar Warts

**DOI:** 10.1155/2022/1220889

**Published:** 2022-11-15

**Authors:** Chen Wu, Xiamin Qiu, Caifeng He, Chao Ci

**Affiliations:** Department of Dermatology, Yijishan Hospital of Wannan Medical College, No. 2 Zheshan West Road, Wuhu, Anhui 241001, China

## Abstract

**Background:**

Plantar warts are a common cutaneous disease of the sole of the foot caused by human papillomavirus. Photodynamic therapy has gained increasing attention in the treatment of plantar warts.

**Objective:**

To investigate the effect of photodynamic therapy combined with transfer factor capsules in the treatment of multiple plantar warts.

**Methods:**

Sixty-one patients with multiple plantar warts who visited our outpatient department from September 2017 to August 2019 were randomly divided into two groups. Twenty-three patients received photodynamic therapy (treatment group) and thirty-eight received cryotherapy (control group). Both groups also received immune modulator transfer factor capsules. Skin lesion score, numeric rating scale- (NRS-) 10 score, recurrence rate, adverse reactions, and Dermatology Life Quality Index (DLQI) were analyzed in both groups.

**Results:**

The mean skin lesion score improved from 13.39 ± 3.88 before treatment to 1.48 ± 2.50 after the last treatment in the treatment group and from 12.47 ± 2.99 before treatment to 4.47 ± 3.67 after the last treatment in the control group. The success rate after 3 months of treatment was 86.96% in the treatment group and 39.47% in the control group. After 3 months of follow-up, the recurrence rate was significantly lower in the treatment group (20%) than in the control group (53.33%). The mean DLQI score at three months after treatment was significantly lower in the treatment group (3.61 ± 1.16) than in the control group (6.31 ± 2.59).

**Conclusion:**

Photodynamic therapy combined with immunomodulators significantly increased the cure rate and reduced the recurrence rate of multiple plantar warts compared with traditional cryotherapy combined with immunomodulators.

## 1. Introduction

Plantar warts are a common cutaneous disease of the sole of the foot caused by human papillomavirus (HPV), with multiple plantar warts defined as more than three plantar warts [1]. The most common symptoms of plantar warts are pain and swelling under the foot, and the annual incidence is 14% [2]. There are many traditional methods for the treatment of plantar warts, including topical application of immunomodulators, local injection of anticancer drugs, and cryotherapy. Topical immunomodulators such as imiquimod exert their effects by enhancing cellular immunity but may not be satisfactory because some patients find long-term treatment difficult to tolerate [3]. Recently, some researchers applied intralesional immunotherapy as the new treatment method for multiple resistant warts, including the intralesional injection of vitamin D3, the tuberculin protein purified derivative, and the measles, mumps, and rubella vaccine [4, 5]. However, larger-scale studies are needed to verify or refute the effect in different populations. Anticancer drugs delivered via local injection, such as pingyangmycin and fluorouracil, act by inhibiting DNA synthesis, but the results may be difficult to achieve because of the strong skin tension on the soles of the feet. Furthermore, this treatment may cause local necrosis and ulcers [6]. In China, cryotherapy with liquid nitrogen is the first-line treatment for plantar warts. However, the main disadvantage of cryotherapy is the high recurrence rate [7, 8].

Photodynamic therapy (PDT) is noninvasive and specific to the target tissue and is mainly used for the treatment of certain actinic keratoses, superficial nonmelanoma skin tumors, photoaging, acne, sebaceous gland hyperplasia, and other hyperplasia as well as inflammatory skin diseases [9–15]. In recent years, PDT has been widely applied in the management of diseases such as condyloma acuminatum and common warts [16]. PDT involves the application of a photosensitizer to the surface of the diseased tissue with active tissue proliferation. Under irradiation with a certain wavelength of light, reactive oxygen species are produced in diseased tissue cells that have absorbed the photosensitizer, resulting in necrosis. Apoptosis is necessary to achieve the purpose of treatment, but the surrounding normal tissues are not affected [17]. To date, the main photosensitizing agent used in PDT is 5-aminolevulinic acid (5-ALA), a low-molecular-weight photosensitizer that readily penetrates the stratum corneum and can be cleared within 24 hours [18].

Transfer factor capsules comprise a mixture of polypeptides, amino acids, and polynucleotides extracted from healthy pig spleen. They may promote the release of interferon, thus selectively stimulating and enhancing the cellular immune response, adjusting the body's immune state, and stabilizing the body environment. They have been used for viral infections and fungal intracellular infections and can also be used as an auxiliary drug with 5-ALA PDT [19].

There are few reports of 5-ALA PDT in the treatment of plantar warts. In the present study, 5-ALA PDT and traditional liquid nitrogen cryotherapy were used to treat multiple plantar warts. Patients were treated with either PDT combined with transfer factor capsules or liquid nitrogen cryotherapy combined with transfer factor capsules. The effectiveness, recurrence, adverse events, and Dermatology Life Quality Index (DLQI) of the two treatments were compared.

## 2. Materials and Methods

### 2.1. Patients

Sixty-one patients with multiple plantar warts who visited the Outpatient Department of Dermatology of Yijishan Hospital, Wannan Medical College, from September 2017 to August 2019 were randomly divided into treatment and control groups. The treatment group comprised 23 patients who received PDT. The control group comprised 38 patients treated with liquid nitrogen cryotherapy. All patients took transfer factor capsules orally (two capsules three times a day).

The inclusion criteria were as follows: no treatment measures such as electric ionization and topical drugs within 2 weeks before enrollment or systemic treatment within 3 months before enrollment; no serious systemic diseases or immune system diseases; age between 18 and 60 years; and having signed the informed consent form.

The following patients were excluded: women who were pregnant or lactating; patients who had systemic immune diseases or had taken oral glucocorticoids and immunosuppressants in the past 6 months; and patients with a history of photosensitivity or cicatricial diathesis.

The study was approved by the Ethics Committee of Yijishan Hospital of Wannan Medical College (IRB approval number 2017-19). All patients provided signed informed consent for study participation prior to receiving PDT or cryotherapy.

### 2.2. 5-ALA PDT

**Figure 1:**
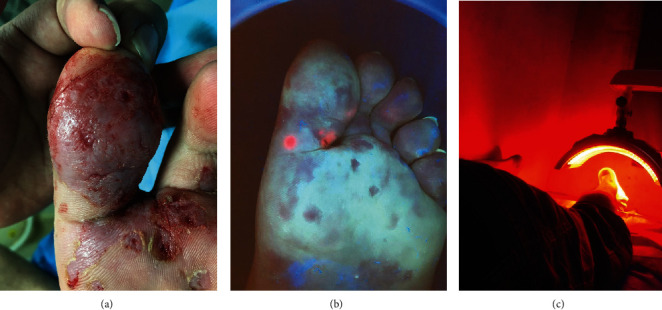
Photographs of a typical case of plantar warts treated with 5-ALA PDT. (a) The preconditioned lesions. (b) PpIX fluorescence observed 3 hours after ALA application. (c) The plantar warts are exposed to a red laser, with the source kept 10 cm from the warts during irradiation.

The thick stratum corneum was scraped off, followed by soaking the feet in white vinegar (5% mass concentration) diluted with warm water (to give a vinegar of 10% volume) for approximately 2 hours. The plantar wart area was covered with 5-ALA photosensitizer (Shanghai Fudan-Zhangjiang Bio-Pharmaceutical Co., Ltd. Shanghai, China) and kept in a black plastic bag for 4 hours. The lesions were then irradiated with 633 nm red light at 80 mW/cm^2^ using a LEDIA PDT instrument (Wuhan Yage Optoelectronics Technology Co., Ltd. Shanghai, China) for 20 minutes. The irradiation area was 18 × 40 cm^2^. The progress of a typical case of plantar warts treated with 5-ALA PDT is shown in Figure 1. After 2 weeks, the decision was made whether to continue treatment on the basis of the condition of the lesions.

### 2.3. Cryotherapy

The feet were soaked in diluted white vinegar and warm water for approximately 2 hours; then, the thick stratum corneum was scraped off; and the skin lesions were disinfected with alcohol. For the cryotherapy, a freezing rod dipped in liquid nitrogen was placed directly on the surface of the plantar warts. The contact time was approximately 15 to 30 seconds to ensure that a 2 mm margin of normal skin around the plantar warts became white. In general, repeated freezing two to five times per session was needed, according to the size of the plantar warts, patient tolerance, and response to cryotherapy. After 2 weeks, the decision was made whether to continue treatment on the basis of the condition of the cryotherapy wound.

### 2.4. Precautions after Treatment

After both types of treatment, patients were given topical iodophor to use to prevent infection. Special treatment was generally not required if blisters and blood blisters appeared following cryotherapy. If there was obvious swelling and pain or the blisters were large, the patient could return to the outpatient clinic for aspiration of blister fluid with a sterile syringe while keeping the blister wall intact. Transfer factor capsules that contained 6 mg polypeptide and 200 *μ*g ribose (Lier Pharmaceutical Co., Ltd, Chengdu, China) were taken orally at a dose of 12 mg three times a day by both groups of patients from the start to the end of treatment.

### 2.5. Data Collection

#### 2.5.1. Efficacy Evaluation

The curative effect was graded as cured (complete clearance), excellent (70%–100% clearance), good (30%–70% clearance), or poor (<30% clearance) at the last treatment [20, 21]. The effective rate was calculated as the number of patients with a curative effect classified as cured or excellent divided by the total number of patients. At present, there is no unified multiple plantar wart symptom score index. The proposed standard after consulting other studies is shown in Table 1 [22, 23].

#### 2.5.2. Recurrence Rate

Recurrence was evaluated on the basis of clinical symptoms. We regarded recurrence to be the appearance of warts on the original skin or adjacent skin within 3 months after the skin lesion was cured. Patients in both groups were followed up at 1, 2, and 3 months after the last treatment, and the recurrence rate was evaluated.

#### 2.5.3. Adverse Reactions

Pain is a common adverse reaction of both PDT and cryotherapy. Pain was assessed using the numeric rating scale- (NRS-) 10 score. In addition to pain, adverse reactions also include burning, scarring, blistering, and other localized skin reactions.

### 2.6. DLQI

The DLQI score was used to indicate the improvement in patient quality of life. This questionnaire measures how much the skin problems have affected the patient's life during the past week. The higher the score, the less satisfied the patient was with the treatment effect [24].

### 2.7. Statistical Analysis

Statistical analysis was performed using SPSS 19.0 software. All data were presented using mean ± standard deviation. The independent *t*-test and *χ*^2^ test were used for the statistical analysis, as appropriate. The difference was considered statistically significant when the *P* value was less than 0.05.

## 3. Results

### 3.1. Comparison of General Information between the Two Groups

The treatment group comprised 23 patients (12 male and 11 female patients), with an average age of 27.36 ± 12.69 years and disease duration of 1.64 ± 0.85 years. The control group comprised 38 patients (12 male and 26 female patients) with an average age of 26.70 ± 7.45 years and disease duration of 1.79 ± 1.10 years. The characteristics of the two groups are shown in Table 2; there were no significant intergroup differences.

### 3.2. Comparison of Skin Lesion Scores and Therapeutic Effects between the Two Groups after Treatment

**Figure 2:**
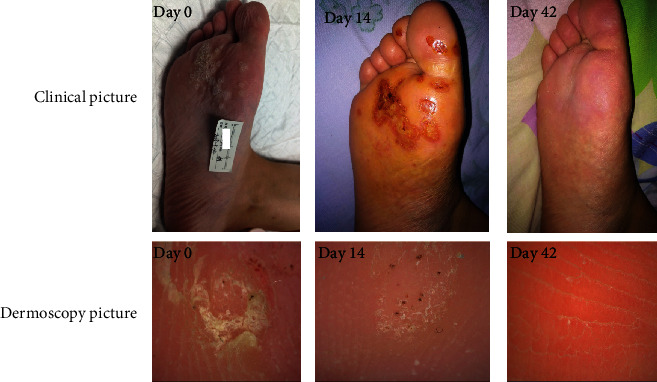
Clinical photographs and dermoscopic images obtained at different time points during the 5-ALA PDT treatment course (days 0, 14, and 42). Plantar warts had a complete response to 5-ALA PDT.

**Figure 3:**
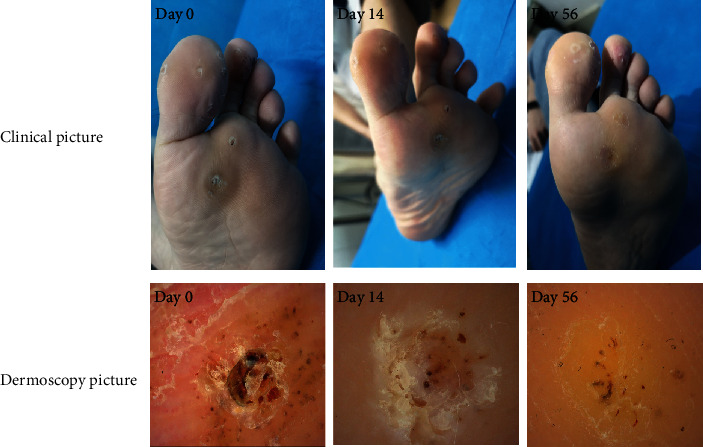
Clinical photographs and dermoscopic images obtained at different time points during the cryotherapy treatment course (days 0, 14, and 56).

Before treatment, the skin lesion scores in the treatment and control groups were 13.39 ± 3.88 and 12.47 ± 2.99 points, respectively. After the third treatment, the skin lesion scores in the treatment and control groups were 4.17 ± 5.36 and 7.42 ± 3.22 points, respectively. After the last treatment, the skin lesion score was significantly lower in the treatment group than in the control group (1.48 ± 2.50 versus 4.47 ± 3.67 points, *P* < 0.05). The effective rate was significantly higher in the treatment group than in the control group (86.96% versus 39.47%, *P* < 0.05). In summary, better effects were observed in the treatment group than in the control group (Table 3 and Figures 2 and 3).

### 3.3. Adverse Reactions

As shown in Table 2, the average NRS-10 pain score was 8.83 ± 2.37 in the treatment group and 4.82 ± 1.39 in the control group after the first treatment. The mean NRS-10 pain score changed to 7.63 ± 0.74 in the treatment group and 2.86 ± 1.42 in the control group after the last treatment. The results indicate that pain was the main adverse effect of PDT treatment. In terms of other adverse effects, swelling was the main manifestation in the treatment group, while blistering was the main manifestation in the control group (Table 3).

### 3.4. Recurrence Rate

Recurrence was observed in four of 23 patients in the treatment group, giving a recurrence rate of 20%, and in eight of 38 patients in the control group, giving a recurrence rate of 46.67%. The *χ*^2^ test showed that the recurrence rate was significantly lower in the treatment group than in the control group (Table 3).

### 3.5. DLQI

In the treatment group, the mean DLQI score 3 months after treatment was 3.61 ± 1.16, which was much lower than that in the control group (6.31 ± 2.59). This result indicates that there was a significant improvement in quality of life after PDT compared with cryotherapy (Table 3).

## 4. Discussion

Plantar warts are benign neoplasms caused by HPV infection on the soles of the feet. Thick skin rashes are often caused by hyperkeratosis at the site of occurrence. General treatments often cannot effectively remove the warts, and there may be subclinical and latent infection that cannot be observed with the naked eye. Multiple and refractory plantar warts are common, and treatment can be a difficult problem for clinicians because of the high recurrence rate. Cryotherapy is the most commonly used treatment to date. Freezing involves rapid crystallization in the wart body via ultra-low-temperature contact with liquid nitrogen. However, the depth and range of freezing are difficult to control and can result in serious adverse reactions. Furthermore, cryotherapy can damage normal tissues and has a high recurrence rate. For example, Bruggink et al. [25] performed cryotherapy on 80 patients with plantar warts; the rate of effectiveness was 46% and the recurrence rate was 53%, with some patients experiencing severe pain, blistering, and hemorrhage. In our study, patients in the PDT and cryotherapy groups were treated up to five times. The average number of treatments in the PDT and cryotherapy groups was 3.78 ± 1.06 and 4.92 ± 0.27, respectively. The total effective rates in the PDT and cryotherapy groups were 86.96% and 39.47%, respectively. The skin lesion scores after the third and last treatments were also significantly lower in the PDT group than in the cryotherapy group. These findings indicate that PDT achieved a significantly better treatment effect than cryotherapy. Furthermore, the recurrence rates in the PDT and cryotherapy groups were 20% and 46.67%, respectively. Therefore, the PDT group also had a higher mean DLQI score than the cryotherapy group. Other studies have reported similar results. Huang et al. [7] investigated 46 patients who received superficial shaving with PDT and 26 patients who received cryotherapy; after 6 months of treatment, the effective rate was 91.3% in the PDT group and 23.1% in the cryotherapy group, and the recurrence rate was 8.7% in the PDT group and 76.9% in the cryotherapy group. Another study that evaluated 121 warts treated with either 20% ALA at 400–700 nm or cryotherapy for 3 weeks reported a cure rate of 75% for ALA PDT compared with 28% for cryotherapy [26].

The photosensitizer used in our study was 5-ALA, which is a low-molecular-weight photosensitizer that can easily penetrate the stratum corneum and can be metabolized by the skin within 24 hours [27]. ALA is the first compound synthesized through the porphyrin-heme pathway and is endogenously converted into the photosensitizer protoporphyrin IX (PpIX). Once PpIX is exposed to its action spectrum (including 400–410 nm and 635 nm), reactive oxygen species are generated, thereby destroying target cells. The light sources of PDT include blue and red light. Blue light includes a spectrum with a wavelength of 405 nm, which can more effectively activate PpIX but cannot penetrate deep tissues because of its relatively short wavelength. Therefore, we often use red light with a wavelength of 635 nm, which can penetrate thick lesions. Beyond the direct phototoxic effects on the target tissues, various cytokines (such as interleukin-1*β* and tumor necrosis factor-*α*) and matrix metalloproteinase-1 are also secreted by fibroblasts during PDT, resulting in immunomodulatory effects in skin disorders [28]. This may be the main reason why PDT can effectively cure plantar warts and reduce the recurrence rate. Wang et al. [29] reported that significant apoptosis in the epidermis 24 hours after the first ALA PDT session was observed by terminal deoxynucleotidyl transferase dUTP nick-end labeling assay, indicating that ALA PDT triggers both apoptosis and necrosis in plantar wart keratinocytes.

In our study, there were adverse reactions in both the treatment and control groups. Previous studies have reported that the most common acute adverse events during and after PDT include pain, erythema, edema, exudation, and crusting [30–32]. However, the main adverse reactions of cryotherapy are pain, blisters, blood blisters, infection, exudation, and edema [33]. In the present study, the mean NRS-10 pain score was higher in the PDT group than in the cryotherapy group, indicating that PDT caused more severe pain during treatment. PDT-induced pain occurs almost immediately after the irradiation. Although the mechanism of PDT pain is not yet clear, the generally accepted view is that inflammation related to cell necrosis interacts with myelinated or unmyelinated nerve fibers. PDT-induced pain is often perceived as a burning sensation that peaks in the first few minutes of treatment. Chaves et al. [34] found that cold spray, intermittent therapy, and local anesthesia with nerve block injection were effective for pain. Warren et al. [35] found that low photodynamic doses (5–10 J/cm^2^) and local anesthetics (such as 3% lidocaine hydrochloride cream) cannot effectively relieve the pain. Miller et al. [36] evaluated 301 patients undergoing PDT and found that 56% of patients did not need pain relief intervention, 35% of patients needed cold spray, and 9% of patients needed to pause the procedure; the study also revealed that larger lesions need more analgesic intervention. However, Ibbotson [37] conducted a meta-analysis of a large number of PDT cases and found that the pain quickly resolves after the irradiation period in most cases. In the present study, we used ice packs and cold spray devices to cool the skin and lidocaine hydrochloride cream to relieve pain during PDT.

As a kind of immunotherapeutic agent, vitamin D has the ability to regulate epidermal proliferation and cytokine production. A previous study found that intralesional vitamin D3 injection achieved better results than Candida antigen and 2% zinc sulfate in the treatment of recalcitrant plantar warts, with a low rate of wart recurrence and minimal adverse effects [38]. In addition, oral vitamin D supplementation has been found to be effective in clearing warts [39]. The transfer factor is a kind of immunomodulator that has been successfully used as an adjuvant in the treatment of intracellular infections such as recurrent herpes virus diseases by improving human immunity. A previous study of 13 women diagnosed with persistent HPV infection who were treated with oral transfer factor plus a cauterizing loop, imiquimod, and podophyllin achieved resolution of their genital lesions without recurrence for at least 1 year [40]. In our study, we used transfer factor as an adjuvant therapy for PDT and cryotherapy.

In summary, PDT combined with immunomodulators increased the curative rate and total effective rate and reduced the recurrence rate of multiple plantar warts. There were no other systemic symptoms except for local painful adverse effects. Our study found that PDT was safe and effective in the treatment of multiple plantar warts and is worthy of promotion.

## Figures and Tables

**Table 1:** Skin lesion scores [22, 23].

Score	Size of lesions	Number of lesions	Degree of skin lesion pain
0	Normal	0-3	No pain
2	Needle tip to millet	4-10	Pain during heavy pressure, no pain during walking
4	Millet to soybean	11-15	Light pressure is pain. It can be tolerated when walking
6	Soybeans to broad beans	16-20	Persistent pain, unbearable walking
8	Broad bean to walnut	More than 21	Persistent pain, cannot stand walking

**Table 2:** Patient demographic data.

Characteristic	ALA-PDT	Cryotherapy	*P*
Gender			
Male	12	12	
Female	11	26	0.11
Age (year)	27.36 ± 12.69	26.70 ± 7.45	0.8
Duration of warts (year)	1.64 ± 0.85	1.79 ± 1.10	0.58

ALA-PDT: aminolevulinic acid photodynamic therapy.

**Table 3:** Patient treatment information.

Characteristic	ALA-PDT	Cryotherapy	*P*
Patients number	23	38	
*Therapeutic effects*			
Number of treatment	3.78 ± 1.06	4.92 ± 0.27	0.001
Curative effects			
Cured	16(69.57%)	9 (23.68%)	
Excellent	4 (17.39%)	6 (15.79%)	
Good	3 (13.04%)	20 (52.63%)	
Poor	0 (0)	3 (7.89%)	0.001
Skin lesion score			
Before treatment	13.39 ± 3.88	12.47 ± 2.99	0.30
After third treatment	4.17 ± 5.36	7.42 ± 3.22	0.001
After last treatment	1.48 ± 2.50	4.47 ± 3.67	0.001
Recurrence			
Yes	4 (20%)	8 (53.33%)	
No	16 (80%)	7 (46.67%)	0.04
Adverse events			
Pain (NRS-10 score)			
After first treatment	8.83 ± 2.37	4.82 ± 1.39	0.001
After last treatment	7.63 ± 0.74	2.86 ± 1.42	0.001
Burning	4 (17.39%)	9 (23.68%)	0.001
Swelling	6 (26.09%)	14 (36.84%)	0.001
Blister	9 (3.91%)	16 (42.11%)	0.001
DLQI			
Before treatment	14.61 ± 2.92	13.74 ± 2.95	0.27
After treatment	3.61 ± 1.16	6.31 ± 2.59	0.001

ALA-PDT: aminolevulinic acid photodynamic therapy; DLQI: Dermatology Life Quality Index.

## Data Availability

The data that support the findings of this study are available from the corresponding author upon reasonable request.
